# P-1924. Impact of the SARS-CoV-2 Pandemic on Medically Attended Acute Gastroenteritis Incidence and Health Care Utilization in the U.S

**DOI:** 10.1093/ofid/ofae631.2084

**Published:** 2025-01-29

**Authors:** Mark A Schmidt, Matthew T Slaughter, John F Dickerson, Holly C Groom, Judy L Donald, Maureen O’Keeffe-Rosetti, Laura H Hendrix, Katherine B Carlson, Emma Viscidi

**Affiliations:** Center for Health Research, Kaiser Permanente Northwest, Portland, Oregon; Kaiser Permanente Northwest Center for Health Research, Portland, Oregon; Kaiser Permanente Center for Health Research, Portland, Oregon; Kaiser Permanente Center for Health Research, Portland, Oregon; Kaiser Permanente Center for Health Research, Portland, Oregon; Kaiser Permanente Center for Health Research, Portland, Oregon; Moderna, Cambridge, Massachusetts; Moderna, Cambridge, Massachusetts; Moderna Therapeutics, Cambridge, Massachusetts

## Abstract

**Background:**

The SARS-CoV-2 pandemic has altered the epidemiology of certain infectious diseases and general utilization of health care services. The goal of this work was to compare the incidence and characteristics of medically attended acute gastroenteritis (MAAGE) prior to and since the pandemic within the Kaiser Permanente Northwest (KPNW) integrated health care delivery system in the US.

**Figure:**
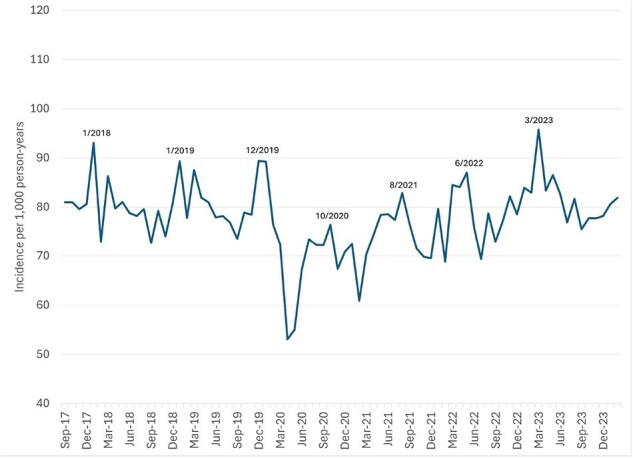

Overall incidence of medically attended acute gastroenteritis among Kaiser Permanente Northwest members, by month, September 2017-February 2024

**Methods:**

We identified MAAGE encounters (remote, outpatient [OP], emergency department [ED] and inpatient [IP]) among KPNW members of all ages, from 9/2017-2/2024, using diagnostic codes in the electronic health record. Encounters separated by < 30 days were considered a single episode and assigned to the highest encounter severity. We included laboratory testing for gastrointestinal (GI) pathogens performed three days prior to three days after MAAGE encounters and reported on norovirus positivity.

**Figure:**
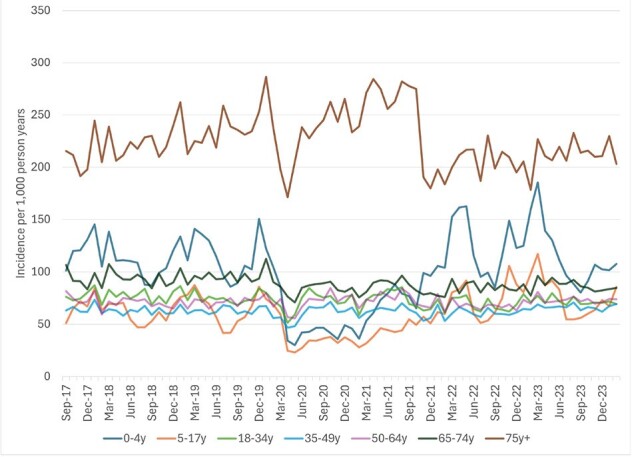

Incidence of medically attended acute gastroenteritis among Kaiser Permanente Northwest members, by age and month, September 2017-February 2024

**Results:**

After a significant decline in early 2020 associated with the start of the pandemic, overall MAAGE incidence rebounded such that the rate observed from 3/23-2/24 (81.6 per 1,000 person years) was similar to that from 3/18-2/20 (80.3 per 1,000 person years; Fig. 1). MAAGE incidence was highest among those 0-4 and 75+ years; however, we observed different patterns between the two periods. Beginning in late 2021, incidence among those 0-4 years returned to, and periodically exceeded, pre-pandemic rates, while that among those 75+ fell below those levels (Fig. 2). Compared to the pre-pandemic era, and since 9/21, a lower proportion of MAAGE episodes had clinical testing for GI pathogens (7.0% vs 8.2%; Fig. 3), a greater proportion were managed remotely (27% vs 15%), and a lower proportion were managed in the OP setting (43% vs. 54%; Fig. 4) p< 0.0001 for all three. We observed no difference in proportions managed in ED (24%) and IP (6%) settings. Among tested episodes, norovirus positivity rebounded since its nadir in 2020.

Figure 3

**Figure:**
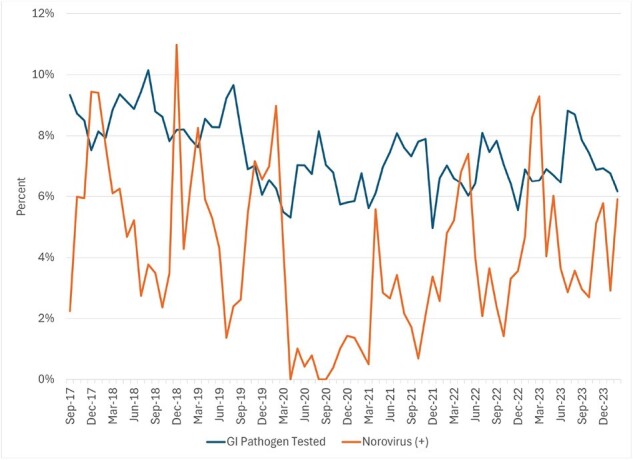

Proportion of overall medically attended acute gastroenteritis episodes for which gastrointestinal pathogen testing was performed, and the proportion positive for norovirus, September 2017-February 2024

**Conclusion:**

Four years after the pandemic began, the epidemiology of MAAGE has largely returned to pre-pandemic levels. At the same time, the shift to greater remote management of MAAGE, with lower rates of diagnostic testing, appears to be sustained. New estimates of the burden of MAAGE on the population and the health care system are required in this changed landscape.

Figure 4

**Figure:**
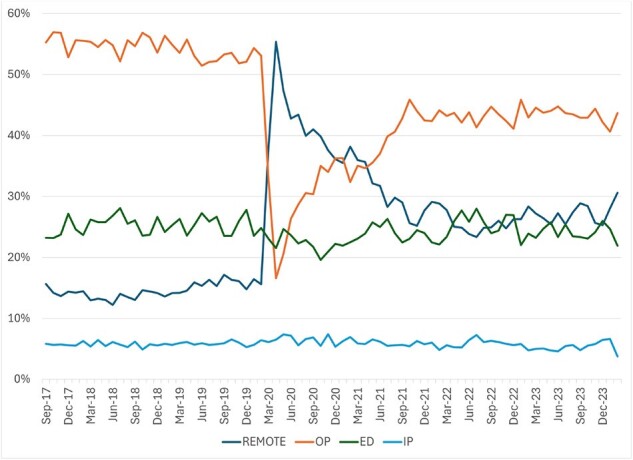

Distribution of medically attended acute gastroenteritis episodes, by location of medical care, September 2017-Feburary 2024

**Disclosures:**

Mark A. Schmidt, PhD, MPH, HilleVax: Grant/Research Support|Janssen: Grant/Research Support|Moderna: Grant/Research Support|Pfizer: Grant/Research Support|Vir Biotechnology: Grant/Research Support Matthew T. Slaughter, MS, Moderna: Grant/Research Support John F. Dickerson, PhD, Janssesn: Grant/Research Support|Moderna: Grant/Research Support|Novartis: Grant/Research Support|Pfizer: Grant/Research Support Holly C. Groom, MPH, Hillevax: Grant/Research Support|Moderna: Grant/Research Support Judy L. Donald, MA, HilleVax: Grant/Research Support|Janssen: Grant/Research Support|Moderna: Grant/Research Support|Pfizer: Grant/Research Support|Vir Biotechnology: Grant/Research Support Maureen O'Keeffe-Rosetti, MS, Hillevax: Grant/Research Support|Janssen: Grant/Research Support|Moderna: Grant/Research Support|Pfizer: Grant/Research Support Laura H. Hendrix, MS, Moderna: Employment|Moderna: Stocks/Bonds (Private Company) Katherine B. Carlson, PhD, MPH, Moderna: Stocks/Bonds (Private Company) Emma Viscidi, PhD, MHS, Moderna: Stocks/Bonds (Public Company)

